# Genetic Characterization of *Staphylococcus aureus* Isolates Associated with Toxic Shock Syndrome Toxin Production: An Epidemiological and Bioinformatics Approach

**DOI:** 10.3390/toxins17090440

**Published:** 2025-09-03

**Authors:** J. R. Aguirre-Sánchez, C. Chaidez-Quiroz, Nohemi Castro-del Campo, Nohelia Castro-del Campo

**Affiliations:** 1 Laboratorio Nacional para la Investigación en Inocuidad Alimentaria (LANIIA), Centro de Investigación en Alimentación y Desarrollo (CIAD), Culiacán 80110, Sinaloa, Mexico; jose.aguirre@ciad.mx (J.R.A.-S.); chaqui@ciad.mx (C.C.-Q.); 2 Facultad de Medicina Veterinaria y Zootecnia, Universidad Autónoma de Sinaloa, Blvd. San Angel 3886, Mercado de Abastos, San Benito, Culiacán 80260, Sinaloa, Mexico; ncastro@uas.edu.mx

**Keywords:** *Staphylococcus aureus*, TSST-1, genomic characterization, virulence markers

## Abstract

*Staphylococcus aureus* is an opportunistic pathogen, a member of the ESKAPE group, associated with nosocomial infections and foodborne illnesses due to its production of various toxins. This study conducted a comprehensive genomic characterization of *S. aureus* isolates producing toxic shock syndrome toxin (TSST-1) using a comparative genomics and bioinformatics approach. A total of 166, including 3 bovine mastitis isolates and 163 public genomes, were analyzed. Twenty-eight distinct sequence types (STs) were identified, with ST30 and ST5 being the most prevalent, corresponding to the clonal complexes CC30 and CC5, respectively. Phylogenetic reconstruction revealed two major clades aligned with these complexes, each exhibiting unique virulence factor profiles. Notably, TSST-1 was detected in bovine mastitis genomes, alongside a broad repertoire of virulence markers, such as enterotoxins and secretion system components, posing a potential risk to public health. Additionally, genes related to environmental information processing systems, including ABC transporters and phosphotransferase systems, were prevalent. These results underscore the need for strengthened genomic surveillance and the implementation of both preventive and corrective measures in dairy herds to mitigate zoonotic transmission and ensure food safety.

## 1. Introduction

*Staphylococcus aureus* is a Gram-positive bacterium recognized as an opportunistic pathogen. It is capable of colonizing a wide range of hosts, including humans, animals, and various environmental niches [1]. As a member of the ESKAPE group, known for high antibiotic resistance and virulence, it is a leading cause of nosocomial infections worldwide [2]. Approximately 30% of the human population is colonized by *S. aureus*, with transmission occurring primarily through direct contact with infected individuals, sick animals, or the consumption of contaminated food [1,3]. Although *S. aureus* is commonly linked to skin and soft tissue infections, it can also cause severe systemic diseases such as bacteremia and pneumonia [4]. The severity of these infections often depends on host factors like immunosuppression. Moreover, *S. aureus* contributes significantly to foodborne infections. The ingestion of food contaminated with enterotoxins may result in acute gastrointestinal disturbances [5]. The dual role as both a pathogenic and a toxin producer makes *S. aureus* a major concern in both clinical and public health settings.

*Sthaphylococcus aureus*’ toxins damage the host through various mechanisms: disrupting membranes, interfering with cellular receptors, or degrading host molecules [6]. Among these, enterotoxins, hemolysis, exfoliative toxins, and toxic shock syndrome toxin-1 (TSST-1) are particularly notable. Enterotoxins and TSST-1 act as superantigens. They induce massive T-cell activation and excessive cytokine release, leading to systemic effects including toxic shock [7]. This “cytokine storm” results in tissue damage, vascular leakage, and multi-organ failure [8,9].

Epidemiological data show that toxin-producing *S. aureus* is responsible for a significant proportion of nosocomial and community-acquired infections [10]. Outbreaks related to food poisoning, toxic shock, and severe skin infections are frequently linked to enterotoxins [11,12] and TSST-1 [9,13,14]. These findings highlight the importance of genomic surveillance in monitoring and controlling virulence.

Particularly, bovine mastitis, primarily caused by *S. aureus*, is characterized by the inflammation of the mammary gland [15]. This represents one of the most prevalent and economically burdensome diseases affecting the dairy industry in Mexico. The economic losses associated with this condition stem from a reduction of up to 30% in both milk yield and quality, increased veterinary treatment costs, premature culling of affected animals, and trade limitations on dairy products. Notably, Mexico ranks fourteenth globally in milk production, with an estimated annual output of 13.721 billion liters, accounting for approximately 2% of the world’s total production [16]. From a public health perspective, bovine mastitis is of particular concern due to the potential transmission of pathogenic microorganisms and contamination with bacterial toxins. Consequently, controlling bovine mastitis is not only a zoonotic priority but also a key strategy for ensuring food safety and consumer health in the country.

In this context, next-generation sequencing (NGS) technologies combined with bioinformatics have become essential tools for detecting toxin-related genes and characterizing the genomic diversity of *S. aureus* [17,18]. These approaches enable high-resolution, genome-wide analyses that surpass traditional methods in both sensitivity and scalability. The application of comparative genomics through NGS facilitates the identification of genetic variations and virulence determinants that contribute to the pathogen’s adaptability and pathogenic potential [19,20]. Moreover, integrating large-scale genomic data with advanced computational analysis allows for phylogenetic relationship reconstruction. This information is critical for improving epidemiological surveillance, refining risk assessments, and designing targeted intervention and control strategies [21]. Ultimately, the synergy between NGS and bioinformatics strengthens the public health framework by enabling more accurate predictions of outbreak dynamics and enhancing the capacity for early detection.

In light of the above, this study aims to genetically characterize *S. aureus* isolates associated with toxin production, particularly TSST-1, using a comparative genomics approach within an epidemiological framework. By identifying virulence-related genes and their genomic context, this study seeks to contribute to the development of timely and robust diagnosis methods, as well as strategies to improve the management and control of *S. aureus* infections.

## 2. Results

**Figure 1:**
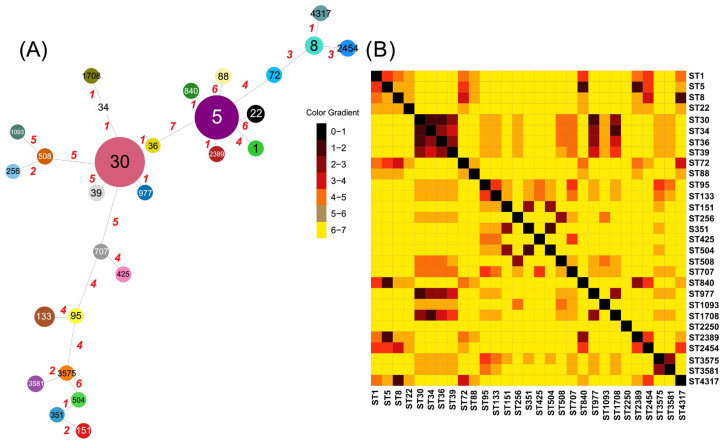
(**A**) *S. aureus* producing the TSST-1 toxin spanning tree. Each colored circle represents a detected sequence type (ST), with its size proportional to the number of identified isolates. The ST number is displayed within the circle. The connecting lines indicate genetic distances based on housekeeping gene comparisons, while the red numbers represent allelic differences. (**B**) Heatmap associated with ST distances. The STs corresponding to each row and column are displayed at the end. The color gradient represents the allelic differences for each comparison. The central black diagonal highlights the comparison of identical STs, resulting in zero difference. Panel (**A**) was created by PHYLOViZ 2.0 and panel (**B**) by Morpheus (https://morpheusdata.com/).

A total of 28 distinct sequence types (STs) were identified among the genomes of *S. aureus* producing toxic shock syndrome toxin (TSST-1) (Figure 1A). ST30 and ST5 emerged as the most prevalent, comprising 54 and 45 isolates, respectively. Despite their high prevalence, these STs do not belong to the same clonal group due to allelic differences in the housekeeping genes analyzed. However, they were found to serve as clonal complexes (CCs) for other STs. Specifically, ST30 was identified as CC30 and as a common ancestor of ST34, ST36, ST39, ST977, and ST1708, whereas ST5, the founder of CC5, includes ST840 and ST2389. These complexes are visible in the distance matrix (Figure 1B), forming regions with intense blue coloration. Additionally, 16 STs were identified as unique to a single isolate.

**Figure 2:**
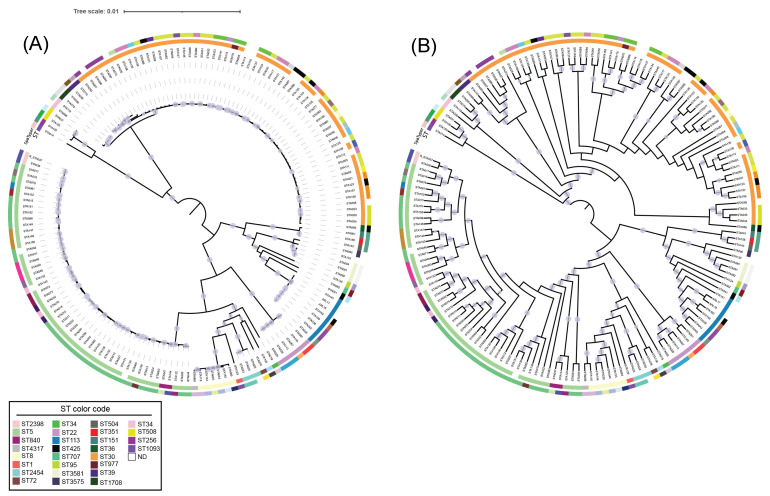
Phylogenetic tree based on 166 genomes of *S. aureus* producing the TSST-1 toxin: (**A**) ML tree with phylogenetic distances. (**B**) ML tree without phylogenetic distances. The gray circles indicate clades with statistical support ≥85. The first outer ring represents the ST identified for each genome, while the second outer ring denotes the SpaTyper classification. The tree was visualized and edited by iTOL V7. The abbreviation ND represents not determined ST.

Regarding phylogeny, the analyzed genomes clustered into two major clades based on their ST classification (Figure 2A). The first clade was predominantly composed of ST5 isolates, while the second mainly consisted of those categorized as ST30. This clustering pattern aligns with the differences observed in allelic profiles. Notably, the ST5 clade exhibited the highest diversity, encompassing 20 distinct STs (20/28), whereas the ST30 clade comprised only 8 STs (8/28) (Figure 2B). Interestingly, ST1093 was identified as the most phylogenetically distant. Additionally, a substantial genetic similarity was observed among members sharing the same ST within each clade, with a genetic variation of only 0.01, as indicated by the tree scale.

**Figure 3:**
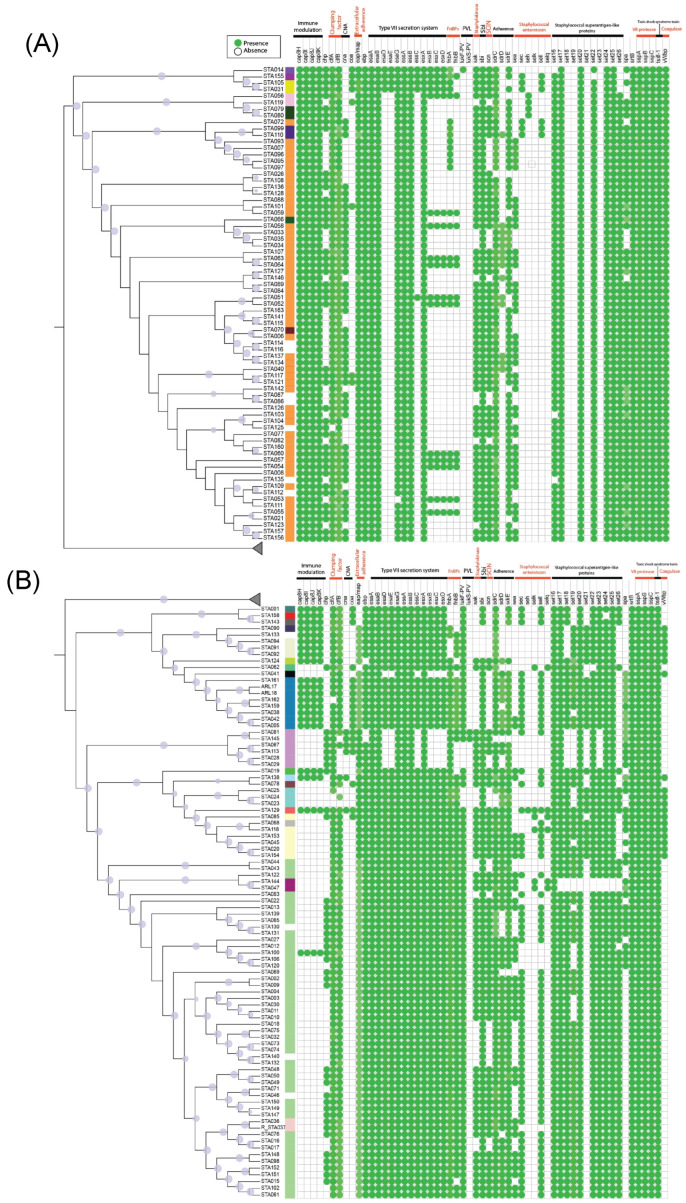
Virulence profile of *S. aureus* genomes. Panels (**A**,**B**) depict a subset of the analyzed genomes. Collapsed clades, representing omitted sections, are indicated by the gray triangles. The upper section displays the virulence genes categorized by their functional annotation. The green circles denote the presence of a virulence gene, while the blank spaces indicate its absence.

Regarding virulence markers, a comprehensive genomic repertoire was identified (Figure 3). The genetic cassette associated with intracellular adhesion proteins responsible for biofilm formation (*icaA-D* and *icaR*) was detected among the homogeneously distributed genotypic elements. The immune modulation operon for the serotype eight capsule and the *lsd* cassette involved in iron acquisition metabolism were also identified. In terms of exotoxins, genes encoding β, α, ϒ, and δ hemolysins were detected, along with the gene responsible for toxic shock syndrome toxin production. Notably, the von Willebrand factor-binding protein (*vWbp*) gene was identified, which encodes a coagulase that facilitates the dissemination of *S. aureus* by promoting thrombotic lesion formation. Moreover, the presence of the V8 protease, associated with the *sspA-C* gene cluster, was also confirmed.

Regarding the capsular genes (Figure 3A,B), we found that most genomes within the clade predominantly composed of ST5—including ST22 and ST2454—lacked the *cap8H-K*, which are essential for capsule biosynthesis. In addition, this group notably lacks the *esaD-E* genes, essential for assembling the type VII secretion system (T7SS). In contrast, the ST30 clade exhibited a distinct genomic repertoire, including five secreted virulence factors (*EsxA-D* and *EssC*), integral components of T7SS, and a characteristic of *S. aureus*. Additionally, ST5 displayed a lower abundance of Staphylococcal superantigen-like (SSL) proteins than other STs. A detailed comparison of virulence markers between genomes isolated from bovine sources and those from human clinical cases revealed a largely homogeneous pattern. Only the *seh*, *sell*, and *selk* genes—associated with enterotoxins—were found to be specific to clinical isolates.

**Figure 4:**
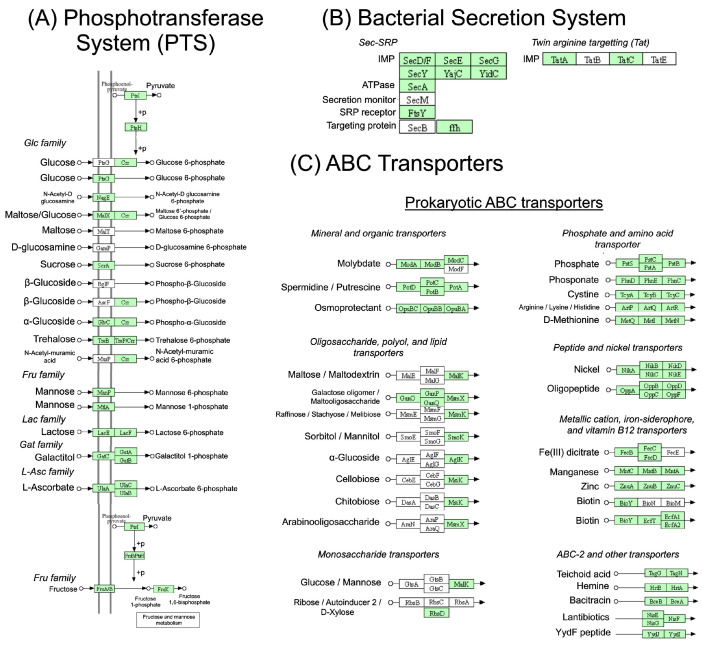
Environmental information processing: (**A**) phosphotransferase system, (**B**) bacterial secretion system, (**C**) ABC transporters. The genes present in the different subsystems are highlighted in green.

Several significant findings were made regarding environmental information processing, which encompasses the phosphotransferase system (PTS), bacterial secretion systems, and ABC transporters (Figure 4). A signal peptide-driven secretion mechanism, characteristic of Gram-positive bacteria such as *S. aureus*, was identified for the bacterial secretion system, facilitating the transport of proteins across the membrane. In the case of phosphotransferases, the study revealed the presence of several enzymes associated with the glucose family, including those responsible for maltose, α-glucosidase, trehalose, and *N-acetyl-D-glucosamine* uptake. Phosphotransferases from other families, such as those involved in lactose, galactitol, L-ascorbate, and fructose transport, were also detected. As for the ABC transporters, a wide array of substrates was identified, including mineral and organic ions, oligosaccharides, phosphates, amino acids, peptides, nickel, metal cations, and members of the ABC-2 and ABCB/C superfamilies.

To sum up, the genomic characterization of *S. aureus* revealed 28 distinct STs, with ST30 and ST5 being the most prevalent. These STs were identified as founders of CC30 and CC5, respectively. Phylogenetic analysis grouped them into two major clades aligned with the ST classification. Virulence profiling demonstrated a consistent presence of genes associated with biofilm formation (*ica* operon), hemolysins (α, β, δ, and ϒ), TSST-1, and other key factors as the *vWbp* gene and the *sspA-C* protease cluster. Differences were noted between components of the T7SS. The environmental information processing systems, including ABC transporters and phosphotransferases were widely presented. The bovine and human isolates showed a largely homogeneous distribution of virulence markers, with the *seh*, *sell*, and *selk* genes found exclusively in clinical strains.

## 3. Discussion

We identified 28 distinct sequence types (STs) among *S. aureus* isolates producing toxic shock syndrome toxin, with ST30 and ST5 being the most prevalent and playing a pivotal role in forming clonal complexes (CCs) CC5 and CC30. In this context, it has been reported that *S. aureus* comprises 100 CCs, with CC5 and CC30 being among the most frequent and strongly associated with hospital-acquired infections [22]. Furthermore, CC30 has been linked to a range of pathologies, including vertebral osteomyelitis, endocarditis, and persistent bacteremia [23,24]. These findings are consistent with molecular epidemiological data reported in other countries. For instance, among the CCs associated with MRSA infections in children in Paraguay, CC5 and CC30 were identified as the two predominant groups [25]. Similarly, studies conducted in Pennsylvania, USA, involving blood infection patient samples, also identified a high prevalence of these CCs, with a particular emphasis on CC5 [26]. On a global scale, numerous studies highlight CC5 and CC30 as the major clonal complexes involved in systemic infections [27,28]. Consequently, molecular techniques, such as sequencing, hold significant promise for enhancing the epidemiological monitoring of this pathogen across different regions. Additionally, the identification of ST30 as CC30 and its relationship with other STs, including ST34, ST36, ST39, ST977, and ST1708, agrees with reports emphasizing the prevalence of ST30 in *S. aureus*-related infections, particularly in regions such as Singapore [29].

Previous studies have identified less prevalent serotypes such as ST1, ST22, ST133, and ST707 in wild animals, including deer, foxes, and reindeer [30]. These findings are consistent with our results, as we were able to detect ST113 in samples collected from cattle with mastitis. This highlights the significant genetic diversity of *S. aureus*, which can complicate efforts for control and prevention. This genetic diversity becomes even more critical, especially in the present context, where all the analyzed genomes harbor genes for the toxic shock syndrome toxin. It underscores the complexity of controlling and preventing *S. aureus* infections, as certain STs may be associated with specific reservoirs.

Regarding the phylogenetic analysis conducted in this study, it was observed that CC5 and CC30 clustered into distinct, independent clades. Evolutionary studies on ST5 have identified an independent evolutionary pathway [31], which may explain its paraphyletic grouping in this study, thereby supporting this evolutionary observation. However, it is essential to consider that the phylogenomic architecture of *S. aureus* is primarily shaped by its clonal nature, horizontal gene transfer events, and recombination [32]. On the other hand, the independent evolution of CC30 has been reported with its speciation and adaptation events closely associated with acquiring virulence markers [33].

The comprehensive genomic analysis of *S. aureus* isolates reveals a diverse and intricate array of virulence markers, which contribute to the pathogen’s pathogenicity and adaptability. One of the most striking findings is the detection of the genetic cassette responsible for intracellular adhesion proteins (icaA-D and icaR), which are crucial for biofilm formation. Biofilms are well-known to enhance bacterial survival in hostile environments, such as the immune system’s defense mechanisms or antibiotic treatment [34]. In addition, biofilms have been found in several human tissues and organs. For example, the colon, stomach, urinary tract, middle ear, and male/female reproductive tract [35,36,37,38]. This genetic repertoire underscores the potential of *S. aureus* strains to form persistent biofilms, making infections difficult to treat and often leading to chronic, recalcitrant infections [3].

Of particular interest is the detection of exotoxins, including the genes encoding β, α, ϒ, and δ hemolysins, alongside the gene responsible for toxic shock syndrome toxin (TSST), further emphasizing the potential severity of *S. aureus* infections. These exotoxins contribute to tissue damage, immune dysregulation, and systemic effects, such as the development of toxic shock syndrome [39,40,41]. TSST is a potent superantigen that induces a hyperactive immune response by stimulating cytokine release, leading to severe systemic effects, including fever, hypotension, and multiorgan failure [42,43]. It is worth highlighting that the *tsst-1* gene was identified in the genomes analyzed in this study. Given that the samples originated from a major dairy herd in northwestern Mexico, it is crucial to implement both preventive and corrective measures to ensure animal health and prevent outbreaks. Such measures should include early detection, the adoption of good livestock management practices, the prompt isolation of infected animals, and the strict application of cleaning and disinfection protocols.

Moreover, von Willebrand factor-binding protein (vWbp) and the SERAMs Coagulase (Coa) are critical elements in blood coagulation induction. This is because coagulases are implicated in thrombotic lesion formation, which enhances the dissemination of *S. aureus* through host tissues [44]. The presence of vWbp and Coa suggests that certain strains may be particularly adept at invading host tissues and spreading throughout the body, thereby exacerbating infection severity [45]. Additionally, identifying the V8 protease, encoded by the *sspA-C* gene cluster, highlights another essential virulence factor. The V8 protease is involved in immune evasion by cleaving host proteins, contributing to the bacterium’s ability to escape immune detection and persist in the host [46].

Aware of the need to contextualize genomic findings, we included genomes previously associated with epidemiological outbreaks and clinical cases, allowing for stronger correlations between genetic virulence determinants and documented clinical manifestations. Specifically, three genomes linked to outbreaks from bakery product consumption in the United States were analyzed [47]. Phenotypic analysis revealed a high biofilm-forming capacity based on absorbance values for bacterial adherence and biofilm formation [48]. The genomic comparison showed similar proportions and identities of the *icaA-D* and *icaR* genes, as well as adhesion-related genes including *clfA-B*, *fnbA*, *ebp*, and *sdrCDE*. A comparable number of genes related to the Type VII secretion system, exotoxins, exoenzymes, and immune modulation were also observed. These findings indicate that our isolates possess significant pathogenic potential, underscoring the importance of preventive and corrective measures in dairy herds to avoid potential outbreaks. Additionally, three genomes from bloodstream infection cases in Brazil and South Korea were included. A comparative analysis revealed a conserved virulence profile between the local and international isolates. Notably, the *can gene* (collagen-binding protein) was exclusively identified in clinical strains. Although it is not considered a primary factor in bacteremia, *can* has been implicated in the binding to collagen-rich tissues such as skin, potentially contributing to diseases like arthritis and endocarditis [49,50].

Our analysis also revealed significant clade-specific differences in the genomic repertoire of *S. aureus* strains. For instance, the ST5 clade, which is associated with severe infections, displayed a unique set of virulence markers, such as the *cap8H-K* genes involved in capsule formation. Interestingly, this clade lacks the *esaD-E* genes necessary for the assembly of the T7SS, which suggests that the virulence of ST5 strains might be mediated through alternative mechanisms. On the other hand, the ST30 clade exhibited a distinct genomic profile, including five secreted virulence factors (EsxA-D and EssC) associated with T7SS, which plays a critical role in the pathogenicity of *S. aureus*. These findings point to the complex and clade-specific nature of *S. aureus* pathogenicity, with different clades relying on distinct sets of virulence factors for successful infection.

Identifying several key mechanisms involved in environmental information processing, such as the PTS, bacterial secretion systems, and ABC transporters, reveals critical aspects of *S. aureus* pathogenicity. The PTS enables the bacterium to efficiently acquire a variety of sugars, supporting its survival and adaptation in different host environments [3]. This metabolic flexibility contributes to its ability to colonize diverse tissues and persist in iron-limited infection sites [3,51]. The bacterial secretion systems, which transport virulence factors like toxins and enzymes, are essential for *S. aureus* to manipulate host immune responses, invade tissues, and form biofilms. For *S. aureus*, this process is instrumental in manipulating host immune responses, invading host tissues, and forming biofilms [52,53].

Additionally, the ABC transporters help the bacterium adapt to nutrient and ion limitations within the host, enhancing its survival and persistence, which may contribute to chronic infections and increased resistance to treatments [54,55]. These findings provide valuable insights into the complex pathogenicity of *S. aureus* and potential therapeutic targets. Understanding these mechanisms offers useful insights into potential therapeutic targets for combating *S. aureus*-related diseases, particularly in the context of emerging antibiotic resistance.

We emphasize that although this research employed bioinformatics and comparative genomics to analyze the presence of genes associated with virulence markers, other emerging technologies may further enhance our understanding of toxin diversity and regulation. In this regard, large-scale functional studies using mutant CRISPR-Cas9 knockout libraries have become pivotal tools for dissecting the biosynthesis and regulatory networks [56]. When integrated with transcriptomic platforms such as BRB-seq (Bulk RNA barcoding and sequencing), it is possible to perform multiplexed gene expression profiling across hundreds of experimental conditions, thereby facilitating the elucidation of regulatory circuits involved in virulence [57]. Additionally, metatranscriptomics approaches offer functional insights into complex microbial communities, allowing the investigation of toxin regulation in natural environments where interspecies interactions can modulate virulence factor expression [58]. Thus, the integration of genomics, transcriptomics, and gene editing analysis provides a comprehensive framework to uncover molecular determinants governing bacterial toxin expression.

## 4. Conclusions

The most prevalent complexes were CC5 and CC30, which also served as founders for other STs. Phylogenetic analysis grouped the isolates into two main clades aligned with these complexes. The presence of TSST and other virulence genes, such as hemolysins and biofilm formation, underscores their role in persistence and pathogenicity. Clade-specific differences in gene content reveal evolutionary divergence and infection potential. The genomes isolated from bovine mastitis cases pose a potential risk to both animal and public health due to the presence of key virulence markers, including the TSST-1 toxin. In this context, it is advisable to investigate genes associated with key virulence markers such as TSST-1, biofilm formation, and exotoxins, which demonstrated a consistent prevalence across the analyzed genomes. Therefore, it is imperative to implement preventive and corrective measures in dairy herds. Additionally, the detection of genes related to environmental information processing, including the PTS, bacterial secretion systems, and ABC transporters, highlights the *S. aureus*’s capacity to thrive in diverse niches and evade host defenses.

## 5. Methodology

### 5.1. DNA Extraction and Sequencing

A total of three confirmed *S. aureus* strains isolated from T3-grade bovine mastitis (from 50 cows) of one of the most important dairy herds in the northwest of Mexico were provided by the National Laboratory for Food Safety Research (LANIIA) at the Centro de Investigación en Alimentación y Desarrollo (CIAD), Culiacán Unit. Genomic DNA was extracted using the DNeasy Blood and Tissue Kit (Qiagen, Hilden, Germany) following the manufacturer’s protocol from single bacterial colonies. The DNA integrity and concentration were assessed with a Qubit dsDNA assay (Thermo Fisher Scientific, Waltham, MA, USA), ensuring a concentration range of 10–50 ng/µL. The extracted DNA was then sequenced at the Earlham Institute using the Illumina MiSeq platform, generating high-quality paired-end reads (forward and reverse) with 100× coverage.

### 5.2. Genomic Characterization by a Bioinformatics Approach

Once sequencing reads were obtained, FastQC [59] was used for an initial quality assessment. Low-quality sequences <50 bp and Phred score <30 were removed using Trim Galore [60]. Additionally, duplicate reads and adapter sequences were filtered to improve data quality. For de novo assembly, the A5-miseq pipeline [61] was implemented, incorporating a scaffold reordering step to optimize the assembly. To conduct a global comparative analysis of toxin-producing *S. aureus* strains, 1996 genomes were retrieved from NCBI and filtered based on the presence of the TSST-1 toxin, yielding 163 genomes for downstream analysis. Multilocus sequence typing (MLST) was performed by comparing allele profiles of the *arcC*, *aroE*, *glpF*, *gmk*, *pta*, *tpi*, and *yqi* genes [62] to determine sequence types (STs) and dominant clonal complexes. A spanning tree was constructed using the PHYLOViZ online tool [63] to visualize the relationships and distribution of STs. The identification of virulence factors was carried out using Abricate V1.0.0 [64] in combination with the Virulence Factor Database (VFDB) [65]. Additionally, spa typing was performed based on polymorphisms in the protein A (*spa*) gene using spaTyper V0.3.3 [66] via command-line execution. For phylogenetic reconstruction, the core genome alignment was performed using Parsnp from the Harvest suite V1.1.2 [67]. A maximum likelihood phylogenetic tree was then inferred using RAxML V8.2.12 [68] under a general time-reversible (GTR) substitution model, with 100 bootstrap replicates for branch support. The resulting tree was visualized, annotated, and midpoint-rooted using iTOL V7 [69].

## Data Availability

The original contributions presented in this study are included in the article. Further inquiries can be directed to the corresponding author.
